# Immunokinetic signatures of *Helicobacter pylori* infection: mechanistic insights for host immunity

**DOI:** 10.3389/fcimb.2026.1937516

**Published:** 2026-09-25

**Authors:** Hiba Orsud, Ahmed Elmontaser Omer Mergani, Abuyaseer Abusaliya, Zakeya Al Rasbi, Merah Abo-Johar, Nura Abo-Johar

**Affiliations:** 1 Microbiology and Immunology, College of Medicine and Health Sciences, United Arab Emirates University, Al Ain, Abu Dhabi, United Arab Emirates; 2 Research Center for Emerging Infections and Zoonoses (RIZ), University of Veterinary Medicine Hannover, Foundation, Hanover, Germany; 3 Institute of Biochemistry, University of Veterinary Medicine Hannover Foundation, Hanover, Germany; 4 College of Dentistry, University of Sharjah, Sharjah, United Arab Emirates

**Keywords:** BabA, CagA, gastritis, *Helicobacter pylori*, host-pathogen, peptic ulcer, SabA, VacA

## Abstract

*Helicobacter pylori* is a highly adaptable bacterial pathogen that affects nearly half of the global population and plays a significant role in chronic gastritis, peptic ulcer disease, and gastric carcinoma. The immunokinetic profile of *H. pylori* infection is characterized by a paradox of cellular response that does not eliminate the bacterium, alongside inconsistent mucosal antibody protection. Reported studies generally demonstrate an early increase in mucosal interleukin-8 (IL-8), followed by increased T helper 1 cells (Th1)- and T helper 17 cells (Th17)-associated cytokine responses, including interferon-γ (IFN-γ), interleukin-17 (IL-17), and tumor necrosis factor-α (TNF-α) Despite these strong proinflammatory responses, the bacterium continues to survive supported by regulatory T cells (Tregs), anti-inflammatory cytokines interleukin-10, transforming growth factor-β1 (IL-10, TGF-β1), and immune-evasion virulence proteins such as VacA (vacuolating cytotoxin A) and CagA (Cytotoxin-associated gene A). Serologically, anti-*H. pylori* IgG is prevalent and long-lived, whereas mucosal IgA responses are variable and generally insufficient for eradication, although they may contribute to partial control of bacterial load. Although these directional trends recur across studies, where cytokine concentrations fluctuate based on sampling methods and patient demographics, clear immunological patterns remain consistent across studies. Active infection is characterized by a generally associated with increased of gastric mucosal IL-8, whereas chronic carriage features a distinct imbalance marked by relatively elevated proinflammatory IFN-γ alongside comparatively modest levels of regulatory IL-10. The imbalance in immunokinetic responses between proinflammatory and anti-inflammatory networks, combined with the genetic diversity of both the host and the pathogen, plays a crucial role in determining clinical outcomes that range from asymptomatic colonization to the development of gastric malignancy. This conceptual synthesis may help identify research priorities and candidate immune patterns for future evaluation, although prospective validation is required before diagnostic, prognostic, or vaccine-related applications can be considered. This review integrates current evidence into an evidence-informed conceptual immunokinetic framework intended to generate testable hypotheses rather than define a universally fixed or clinically validated sequence of immune events.

## 1 Introduction

*Helicobacter pylori* is a spiral shaped, Gram negative, microaerophilic bacterium that colonizes the human gastric mucosa and represents a model of chronic bacterial infection with major implications for mucosal immunology and gastric cancer biology (Amieva and Peek, 2016). It is recognized as the first bacterial carcinogen and is causally linked to chronic gastritis, peptic ulcer disease, gastric adenocarcinoma, and gastric mucosa associated with lymphoid tissue lymphoma (Amieva and Peek, 2016; Kusters et al, 2006). From a global health perspective, the infection demonstrates how microbial transmission patterns are shaped by sanitation, socioeconomic development, and antibiotic exposure. At the mucosal level it exemplifies a pathogen capable of eliciting strong innate and adaptive responses without achieving clearance, giving rise to a persistent inflammatory environment that may progress to malignancy (Amieva and Peek, 2016; Suerbaum and Michetti, 2002). Despite extensive literature describing the innate and adaptive immune responses associated with *H. pylori* infection, most reviews present these pathways as relatively static and compartmentalized events. However, increasing evidence suggests that *H. pylori* persistence is fundamentally governed by dynamic temporal interactions between epithelial sensing, inflammatory amplification, immune regulation, and progressive immune exhaustion. In this review, we propose an immunokinetic framework that conceptualizes *H. pylori* infection as a continuously evolving host–pathogen equilibrium rather than a fixed chronic inflammatory state. In this review, we integrate current evidence into a conceptual immunokinetic framework intended to generate testable hypotheses regarding the temporal and spatial organization of host–pathogen interactions during *H. pylori* infection. The proposed framework is an evidence-informed conceptual model rather than a prospectively validated sequence of immune events. However, the term “*immunokinetic signature”* in this review refers to the temporal and dynamic progression of the host’s immune response throughout *H. pylori* infection. Instead of examining specific cytokines or immune cells, the immunokinetic signature integrates sequential changes in innate immunity, adaptive immunity, inflammatory mediators, bacterial virulence factors, and disease progression (**Figure 1**).

**Figure 1:**
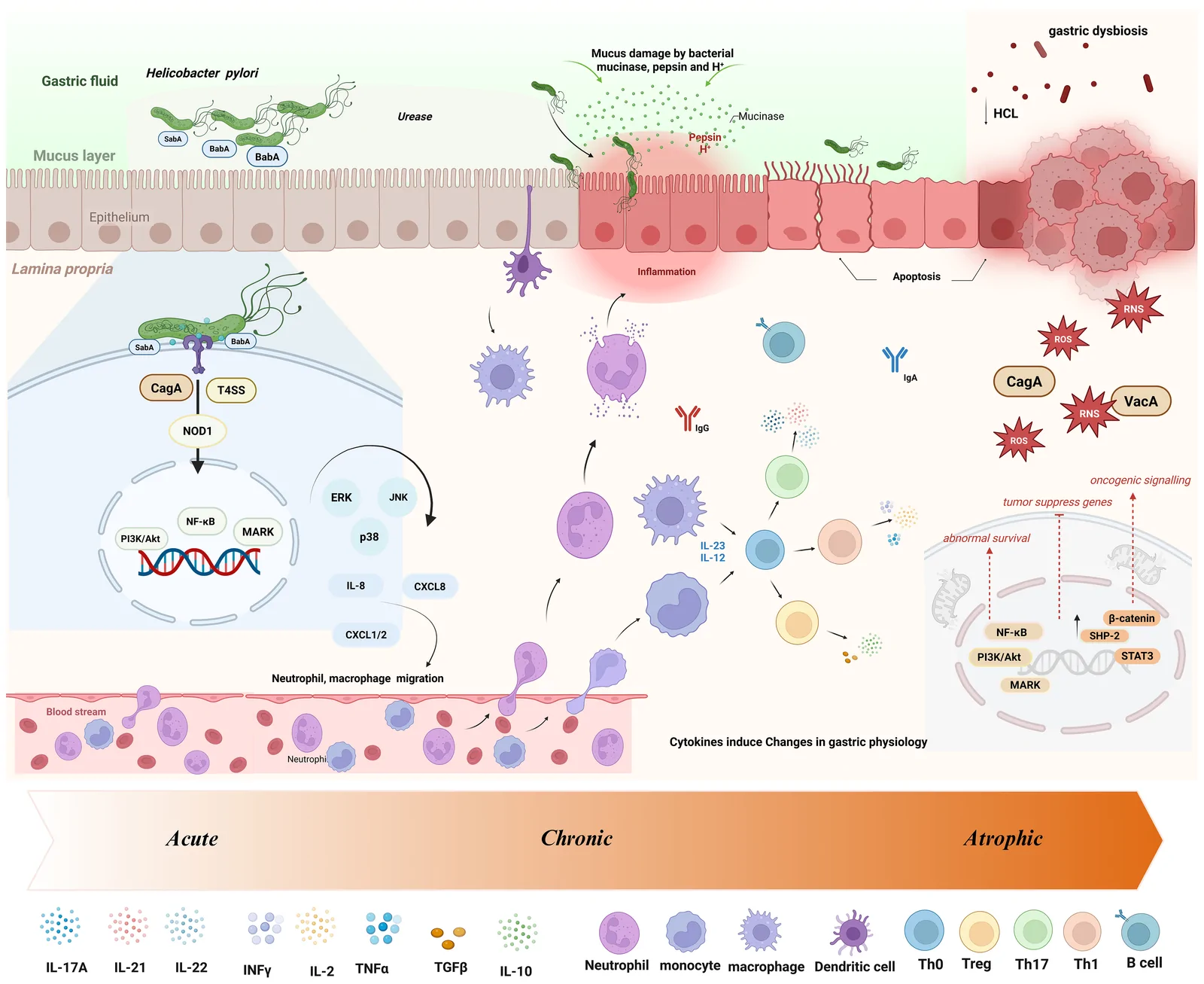
Summarizing the conceptual immunokinetic framework of host–pathogen interactions during *H. pylori* infection: Following colonization of the gastric mucus layer, *H. pylori* adheres to epithelial cells via BabA and SabA adhesins and releases urease, mucinase, and other enzymes that disrupt the mucosal barrier. Delivery of CagA through the T4SS activates intracellular NOD1 signaling in epithelial cells, triggering NF-κB, MAPK (ERK, JNK, p38), and PI3K/Akt as protective pathways and promoting the production of chemokines such as IL-8/CXCL8 and CXCL1/2. These mediators recruit neutrophils and macrophages to the lamina propria, initiating acute gastritis. As infection persists, cytokine networks drive the transition to chronic inflammation characterized by Th1- and Th17-skewed responses (IFN-γ, IL-17A, IL-21, IL-22, TNF-α) alongside regulatory mechanisms mediated by IL-10, TGF-β, and Tregs expansion. Antibody production (IgA, IgG) develops but does not eradicate infection. Sustained epithelial injury, apoptosis, and inflammatory signaling alter gastric physiology and promote microbial dysbiosis. In long-standing infection, persistent NF-κB activation and oxidative stress (ROS/RNS), combined with CagA- and VacA-mediated signaling, promote oncogenic pathways engagement, including β-catenin, STAT3, MAPK, and PI3K/Akt activation, along with suppression of tumor-regulatory mechanisms. These changes support abnormal epithelial survival, glandular loss, and progression toward atrophic gastritis, intestinal metaplasia, and gastric carcinogenesis. The diagram is an evidence-informed conceptual synthesis and does not indicate a universally fixed sequence, validated time scale, causal hierarchy, or inevitable progression toward atrophy or malignancy.

### 1.1 Global impact and epidemiological context

A large systematic review including data from 62 countries estimated that in 2015, approximately 4.4 billion individuals were infected, representing more than half of the global population (Hooi et al., 2017). Regional prevalence varies considerably, with Africa and Latin America consistently exceeding sixty to seventy percent, in contrast to Western Europe and North America where prevalence is roughly one third (Zamani et al., 2018; Eshraghian, 2014; Alsulaimany et al., 2020). Although improvements in sanitation, access to safe water, and widespread eradication therapy have contributed to declining prevalence in high income countries, *H*. *pylori* remain highly prevalent in many low- and middle-income settings (Eusebi et al., 2014; Kotilea et al., 2019). Infection is usually acquired in childhood through intrafamilial oral or fecal routes and persists in lifelong unless treated (Balas et al., 2022). Global and regional prevalence is summarized in **Table 1**. Overall, the GCC demonstrates intermediate prevalence pattern with substantial inter-country variation that may shape host–pathogen interactions (Orsud et al., 2020; Al-Shamsi et al., 2022; Chen et al., 2024).

**Table 1:** Global prevalence of *H. pylori* infection.

Region	Latest estimated prevalence (%)	Representative studies
Africa	70.0–78.1	Hooi et al., 2017; Kusters et al., 2006
South-East Asia	65–75	Hooi et al., 2017
Europe	30–45	Malfertheiner et al., 2022
North America	25–35	McColl, 2010
Gulf Region (GCC)	50–65	Al-Shamsi et al., 2022
Eastern Mediterranean	48.7–56.6	Chen et al., 2024

## 2 Pathogenesis and immunobiological paradox

Successful colonization of *H. pylori* requires acid adaptation, motility, adhesion, and immune modulation (Salama et al., 2013). Urease activity generates ammonia that buffers local acidity, enabling survival in the gastric mucosa (Kusters et al, 2006). Motility driven by flagella allows navigation through mucus to the epithelial surface, where adhesins such as BabA (Blood group antigen–binding adhesin) and SabA (Sialic acid–binding adhesin) maintain stable attachment (Amieva and Peek, 2016; Yamaoka, 2010). These early colonization steps and epithelial interactions are illustrated in **Figure 1**. One of the key virulence factors include the cytotoxin-associated gene A (*cagA*) pathogenicity island which encodes a type IV secretion system (T4SS) capable of injecting CagA into gastric epithelial cells, as well as the vacuolating cytotoxin VacA (Polk and Peek, 2010). CagA alters host signaling pathways including Src homology two domains of the phosphatase (SHP2), mitogen-activated protein kinase (MAPK), and β-catenin, whereas VacA disrupts mitochondrial function, endosomal trafficking, and T cell activation (Polk and Peek, 2010). Their downstream effects on epithelial injury and immune signaling are depicted in **Figure 1**. CagA indirectly disrupts the innate immunity by hijacking host signaling pathways that intersect with innate immune responses. Innate immune recognition occurs primarily via Toll like receptors 2, 4, and 5, and intracellular nucleotide-binding oligomerization domain-containing protein 1(NOD1) sensing of bacterial peptidoglycan. Activation of nuclear factor NF kappa B leads to secretion of interleukin-8 (IL-8) and other chemokines that recruit inflammatory cells (Salama et al., 2013). Despite vigorous innate activation the bacterium evades clearance by modifying lipopolysaccharide (LPS) structure, altering antigenic exposure, and interfering with macrophage killing pathways (Blaser and Atherton, 2004).

Adaptive immunity is dominated by T helper type 1 (Th1) and T helper type 17 (Th17) responses which contribute to inflammation but fail to eradicate the infection, because regulatory T cells (Tregs) have been observed to expand in the gastric mucosa, produce interleukin-10 (IL-10) and transforming growth factor-β1 (TGF- β1), and suppress effector responses, promoting bacterial persistence (Arnold et al., 2011). This coexistence of inflammation and immune regulation -central to the *H. pylori* immune paradox- is summarized in **Figure 1**. Furthermore, VacA further skews dendritic cell maturation toward a semi-tolerogenic phenotype that reduces antigen presentation (Blaser and Atherton, 2004). The coexistence of strong inflammation and continued bacterial survival represents the core immunological paradox of *H. pylori* infection (Ruggiero, 2010). Importantly, conventional models describing *H. pylori* pathogenesis frequently interpret inflammatory and regulatory responses as parallel but independent processes. However, emerging evidence indicates that these responses are temporally interconnected and evolve progressively throughout infection. Early neutrophilic and Th1/Th17-dominant responses initially restrict bacterial expansion but simultaneously establish the inflammatory conditions that favor subsequent regulatory dominance, T-cell exhaustion, and chronic persistence. This temporal transition may explain why robust immune activation fails to achieve sterilizing immunity despite sustained cytokine production and dense inflammatory infiltrates. The immune skew toward Tregs populations strongly influences disease phenotypes, including ulceration and carcinogenic potential (Sgouras et al., 2015). The cumulative epithelial damage, immune activation, and regulatory imbalance shown in **Figure 1** underpin the progression from chronic gastritis to ulceration and neoplastic transformation (Yuan et al., 2025).

## 3 Clinical spectrum and disease outcomes

The natural history of *H. pylori* infection in the absence of therapeutic intervention; is characterized by host immunological pathways, bacterial virulence factors, and gastric tissue outcomes. The temporal progression from acute infection to chronic symptomatic, chronic asymptomatic, and atrophic/precancerous states is summarized in (**Table 2**), which highlights dominant host signaling pathways, key bacterial factors identified by the host, associated alterations within the gastric microenvironment, and prevalent clinical and biomarker results outcomes.

**Table 2:** *H. pylori* infection progression from acute to chronic and atrophic phases and pathways.

Phase	Dominant pathways	Key bacterial factors	Tissue/outcome	Supporting studies
Acute	NF-κB, MAPK, NOD1	T4SS, CagA, Urease, VacA	Neutrophilic gastritis	Fu et al., 2025; Niu et al., 2020
Chronic asymptomatic	Tregs, IL-10/TGF-β, Checkpoint pathways	VacA, LPS variants	Low-grade inflammation	Villarroel-Espindola et al., 2023
Chronic symptomatic	Th1/Th17, ROS/RNS, COX-2	CagA, VacA, BabA/SabA	Active chronic gastritis	Sah et al., 2023; Yang et al., 2021
Atrophic/precancerous	NF-κB, STAT3, β-catenin, PI3K/Akt	*cagA+*, VacA	Atrophy → Metaplasia → Cancer	de Souza et al., 2022; Díaz et al., 2018

### 3.1 Acute infection

After acquisition, typically within days to weeks, *H. pylori* establishes colonization of the gastric mucosa and triggers an acute inflammatory response. Pattern-recognition receptors (PRRs) detect bacterial components, while NOD1 senses peptidoglycan delivered through the T4SS. Which activates downstream nuclear factor kappa B (NF-κB) and MAPK pathways (ERK, p38, JNK), leading to robust induction of IL-8/CXCL8 and related chemokines (CXCL1/2) (Jang et al., 2026). These signals promote neutrophil recruitment and initiate early Th1/Th17 immune priming (Fu et al., 2025). The resulting neutrophil recruitment contributes to acute gastritis and epithelial injury. However, simultaneous immune-evasion mechanisms allow the organism to persist. Histologically, acute infection is associated with active neutrophilic gastritis, epithelial stress; such as exposure to oxidative stress, and early disruption of the mucus barrier. Clinically, this phase may be asymptomatic or accompanied by transient dyspepsia, with elevated IL-8 levels and positive fecal antigen or urea breath tests once colonization is established (Niu et al, 2020; Senchukova, 2022).

### 3.2 Chronic infection

As infection persists over months to years. many infected individuals progress into a chronic asymptomatic state that may persist for years to decades. This phase is characterized by a shift toward immune regulation, with Tregs predominance and context-dependent changes in regulatory mediators, including IL-10 and TGF-β1. The direction and magnitude of these changes may vary according to bacterial genotype, biological compartment, disease context, and analytical method (Salavati et al., 2020; Zhu et al., 2025; **Table 3**). In addition to the immune checkpoint-mediated suppression and macrophage polarization toward regulatory phenotypes consistent with the established immunoregulatory role of macrophages (Villarroel-Espindola et al., 2023; Mosser and Edwards, 2008). However, macrophage responses during *H. pylori* infection should not be viewed as a simple transition from proinflammatory to regulatory phenotypes. Gastric macrophages are exposed simultaneously to bacterial products, epithelial-derived mediators, Th1/Th17 cytokines, and regulatory signals, potentially generating heterogeneous or overlapping activation states. The relative contribution of these macrophage populations to bacterial control, tissue injury, immune tolerance, and progression toward neoplasia remains incompletely defined. Single-cell and spatial profiling of human gastric tissue across disease stages will be required to resolve this heterogeneity.

**Table 3:** Reported cytokine trends and study-specific concentration ranges during *H. pylori* infection.

Cytokine	Functional role	Reported direction of the measured cytokine outcome’s	Specimen/biological compartment	Assay platform or analytical method	Phase of infection	Representative Sources
IL-8	Neutrophil chemoattractant	variable: Circulating IL-8 was increased in *H. pylori*-positive, but *IL-8* expression decreased in gastric tissue.	Plasma/gastric biobsy	Multiplex ELISA for plasma; RT-qPCR for gastric tissue	Acute/Early	Saaed et al. (2021); Yang et al. (2024)
IFN-γ	Th1 activation, macrophage priming	Increased: *H. pylori*-infected gastric mucosa demonstrated increased IFN-γ expression and greater infiltration by IFN-γ-producing T cells	Gastric mucosal biopsies	functional cell-culture assays: RT-qPCR, western blot, ELISA	Chronic active gastritis	Kong et al. (2020).
IL-17A	Th17 response, epithelial recruitment	Increased IL-17-related measures in peripheral blood and gastric mucosa during *H. pylori*-positive gastritis	Serum; gastric biobsy	Luminex for serum IL-17A; functional cell-culture assays: RT-qPCR, immunohistochemistry, ELISA	Chronic gastritis	Della Bella et al. (2023); Zhu et al. (2025)
IL-10	Anti-inflammatory regulation	Increased but context-dependant	Gastric biobsy; peripheral blood	Immunofluorescent, RT-qPCR, ELISA	Persistent	Della Bella et al. (2023); Zhu et al. (2025)
TGF-β1	Treg-mediated tolerance	variable/context-dependant: A recent study discovered elevated levels of TGF-β1 in the gastric mucosa and peripheral blood. However, in an earlier infected-only cohort, there was a little increase in TGF-β1 expression among *cagA*-positive cohort	Gastric biobsy; peripheral blood, mucosal tissue	RT-qPCR, immunohistochemistry, ELISA, mucosal proteomics	Persistent	Salavati et al. (2020); Zhu et al. (2025)
TNF-α	Amplifies mucosal injury	Increased: TNF-α was significantly higher in the peptic ulcer patients.	Gastric biobsy	RT-qPCR, immunohistochemistry, ELISA	Chronic active gastritis and peptic ulcer disease	Rahimian et al. (2022); Yang et al. (2024)

Although Th1 and Th17 cells remain detectable, their effector functions are restrained, and acute neutrophilic influx is markedly reduced (Mohammadzadeh et al., 2023). At this asymptomatic stage, Low-grade *NF-κB* and *MAPK* signaling persists, reflecting ongoing host–pathogen coexistence rather than resolution. Importantly, the bacterial virulence repertoire often remains intact; however, host regulatory mechanisms supported by VacA activity, LPS modifications, and Tregs induction limit tissue damage while permitting long-term persistence of *H. pylori*. Histologically, the gastric mucosa shows low-grade chronic inflammation with gradual remodeling, and in some individuals, particularly those with corpus-predominant gastritis, progression toward atrophy of gastric mucosa may occur. Clinically, patients are often asymptomatic, yet remain at risk for atrophic gastritis, intestinal metaplasia, and malignancy over time, depending on bacterial strain, virulence factors, host genetics, and environmental exposures, such as socioeconomic condition (Fu et al., 2025; Senchukova, 2022).

In contrast, a subset of individuals develops chronic symptomatic disease characterized by active chronic gastritis and persistent dyspeptic symptoms (Kim, 2024). This phase reflects sustained inflammatory signaling, with prolonged activation of NF-κB and MAPK pathways and maintenance of adaptive Th1 and Th17 responses, including interferon-γ (IFN-γ), interleukin-17A and Interleukin-17F (IL-17A/F), and IL-22 (Dewayani et al., 2021), Recent primary studies generally report increased gastric mucosal tumor necrosis factor-α (TNF-α) expression during chronic active gastritis and peptic ulcer disease, although the magnitude of the increase varies among patient cohorts and analytical methods (Rahimian et al., 2022; Yang et al., 2024). Inflammasome activity - cytosolic pattern-recognition complexes which bridge between epithelial stress and chronic immune activation - may contribute variably, while ongoing production of reactive oxygen and nitrogen species drives epithelial injury and remodeling of tight junctions and barrier integrity (de Souza et al., 2022). Upregulation of prostaglandin synthesis via cyclooxygenase (COX-2) further amplifies inflammation and pain signaling (Guardado et al., 2025). At this stage, *H. pylori* virulence factors such as CagA mediating SHP-2/ERK signaling and β-catenin dysregulation VacA-induced T-cell suppression and mitochondrial dysfunction, adhesins BabA and SabA, and modified LPS structures play central roles in sustaining inflammation while evading immune clearance (Sundqvist et al., 2023). As mentioned earlier, the gastric niche exhibits active chronic gastritis with mononuclear cell infiltration and intermittent neutrophil presence, with an increased risk of erosions or peptic ulcers depending on acid output, bacterial strain, and host factors. Biomarker profiles studies generally report elevated mucosal IL-8, IFN-γ, and IL-17, and histological examination typically confirms chronic active gastritis (Sah et al., 2023; Yang et al., 2021).

### 3.3 Chronic atrophic/precancer track

With prolonged infection, a subset of patients enters a chronic atrophic or precancerous trajectory, which may also remain clinically silent. This stage is driven by epithelial transformation pathways rather than classic inflammatory responses. Chronic NF-κB activation combined with sustained oxidative stress promotes engagement of oncogenic signaling cascades, including β-catenin, STAT3, EGFR/MAPK, and PI3K/Akt pathways, alongside epigenetic silencing of tumor suppressor genes. Progressive loss of acid-secreting glands leads to hypochlorhydria, alterations in the gastric microbiome, and features of immune exhaustion or tolerance (Fu et al., 2025; Mohammadzadeh et al., 2023; Linares et al., 2023). ***c****agA* -positive *H. pylori* strains are frequently associated with this trajectory due to their enhanced oncogenic signaling capacity and ability to sustain long-term epithelial perturbation. In histopathological, this phase is marked by glandular atrophy and intestinal metaplasia, with corpus involvement being particularly relevant. Clinically, patients face an increased risk of progression from intestinal metaplasia to dysplasia and gastric adenocarcinoma, and may develop micronutrient deficiencies such as vitamin B12 or iron deficiency as gastric function deteriorates (Farinati et al., 2008; Díaz et al., 2018).

However, this progression should not be interpreted as an inevitable linear sequence. Only a minority of infected individuals develop gastric malignancy despite prolonged colonization, indicating that bacterial virulence alone or persistent inflammation alone is insufficient to explain carcinogenesis. A plausible model is that cancer risk emerges from the cumulative interaction of bacterial genotype, host inflammatory susceptibility, immune-regulatory failure, epithelial alterations, microbiome changes, and duration of exposure. Prospective studies tracking these variables before the development of premalignant lesions are needed to distinguish causal drivers from biomarkers that merely accompany disease progression.

Most infected individuals remain asymptomatic for many years, although virtually all develop histological gastritis (Sugano et al., 2015). Clinical outcomes range from non-ulcer dyspepsia to peptic ulceration, gastric adenocarcinoma, and gastric mucosa associated lymphoid tissue lymphoma. Peptic ulcer disease occurs in approximately ten to fifteen percent of infected individuals (Kusters et al, 2006). Eradication therapy significantly diminishes the risk of ulcer recurrence and hemorrhage, modifying the natural progression of peptic ulcer disease (Malfertheiner et al., 2022). Chronic *H. pylori* gastritis is the major cause of non-cardia gastric adenocarcinoma worldwide and is also linked to gastric mucosa associated lymphoid tissue lymphoma (Amieva and Peek, 2016; Uemura et al., 2001). Between eighty and ninety percent of non-cardia gastric cancers are attributable to chronic infection (48). Although only one to three percent of infected individuals progress to cancer, the high global prevalence makes *H. pylori* a leading infectious cause of malignancy (Kusters et al, 2006; Plummer et al., 2015).

Host cytokine gene polymorphisms, including variants in *IL1β*, *TNFα*, and *IL10*, modulate the magnitude of the inflammatory response and influence susceptibility to gastric carcinogenesis (Salama et al., 2013). Concurrently, bacterial virulence determinants such as *cagA, vacA* allelic types, and adhesins including BabA and SabA modulate colonization and epithelial damage (Yamaoka, 2010; Polk and Peek, 2010). Persistent immune activation leads to oxidative stress, DNA damage, epithelial remodeling, glandular atrophy, and intestinal metaplasia, establishing a microenvironment conducive to dysplasia and carcinoma (Polk and Peek, 2010). Regulatory pathways involved in bacterial persistence may also contribute to local T cell exhaustion and immune escape in late-stage lesions (Blaser and Atherton, 2004).

The remarkable variability in disease outcomes highlights that pathogenesis is influenced not only by infection status however by the dynamic interplay between bacterial virulence and host immune control across time. This underlines how important it is to investigate *H. pylori* infection via an immunokinetic paradigm that reflects the evolving characteristics of mucosal immune responses. To address these limitations, this review proposes an immunokinetic framework that integrates temporal immune responses, cytokine kinetics, bacterial adaptation mechanisms, and disease outcomes into a unified model of *H. pylori* persistence and pathogenesis.

## 4 Evidence-informed conceptual immunokinetic framework

The following framework distinguishes observations supported by published experimental or clinical studies from integrative relationships proposed in this review. Unless a temporal or causal connection has been directly demonstrated, it is presented as a conceptual inference that requires prospective validation. Within this conceptual framework, *H. pylori* infection is viewed as a process involving overlapping phases of immune activation, regulation, bacterial adaptation, and tissue remodeling. These phases are not assumed to occur in an identical sequence or at fixed intervals in all patients. Available evidence suggests that epithelial and innate immune activation generally becomes evident early, whereas adaptive and regulatory responses become increasingly prominent as infection persists; however, substantial overlap and interindividual variability are expected. During the early phase of colonization, gastric epithelial cells and resident myeloid cells detect the bacterium through PRRs expressed on epithelial cells, macrophages, and dendritic cells (DCs) within the gastric mucosa and mount an acute inflammatory response (Cheok et al., 2022). As infection persists, this innate phase transitions into a predominantly Th1- and Th17-skewed adaptive response, accompanied by a more modest Th2 component, and may subsequently become more prominent of Tregs and maturation of humoral immunity (Alzahrani et al., 2024; Wu et al., 2025). Under normal physiological conditions, Th1/Th2 cells are in a dynamic equilibrium, but Th1/Th2 immune imbalance induced by *H. pylori* infection is a key factor driving the pathological process thereby shifting the Th1/Th2 balance towards immune tolerance (Wu et al., 2025). Despite sustained cytokine production and dense cellular infiltrates, bacterial loads usually reach a plateau instead of declining to zero, reflecting a stable but pathogenic equilibrium between host immunity and bacterial adaptation (Cheok et al., 2022; Dixon et al., 2019).

Despite decades of investigation, several fundamental questions in *H. pylori* immunobiology remain unresolved. One of the central paradoxes is why a vigorous Th1- and Th17-dominant inflammatory response consistently fails to eradicate the organism. Although inflammatory cytokines such as IFN-γ, TNF-α, and IL-17 contribute to bacterial restriction, they also promote epithelial injury, oxidative stress, and tissue remodelling that may indirectly favor bacterial persistence. Another unresolved issue concerns the precise role of regulatory immunity. While Treg-mediated IL-10 and TGF-β1 production clearly limits tissue damage, excessive regulatory activity may simultaneously suppress protective antibacterial responses and facilitate chronic colonization. Similarly, accumulating evidence suggests that mucosal IgA responses may exhibit dual functionality, contributing both to immune exclusion and, paradoxically, to bacterial persistence by shielding *H. pylori* from complement-mediated clearance.

Importantly, most available studies examine isolated immune pathways rather than integrated temporal immune transitions. As a result, the field still lacks a unifying mechanistic model capable of explaining how inflammatory activation, immune regulation, epithelial adaptation, and bacterial immune evasion collectively evolve during long-term colonization. Addressing these unresolved questions is essential for the development of biomarker-guided diagnostics, precision immunotherapies, and effective mucosal vaccines.

### 4.1 Early innate recognition and epithelial signaling

Gastric epithelial cells express a repertoire of PRRs that are central to the earliest phase of *H. pylori* sensing. Toll like receptors 2 and 4 (TLR-2 and 4) recognize atypical LPS structures and other outer membrane components, while the cytosolic sensor (NOD1) detects peptidoglycan fragments that gain access to the cytoplasm, often via the *cag* type four secretion system (Ruggiero, 2010). Engagement of these receptors activates NF-κB and activator protein 1 signaling pathways and leads rapidly to transcription of pro inflammatory mediators such as IL-8, IL-1β, TNF-alpha, and IL-6 (Cheok et al., 2022; Sharma et al., 1998). *In vitro* studies using gastric epithelial cell lines and primary cultures show that exposure to live *H. pylori* induces a strong and early increase in *IL8* gene transcription and secretion, particularly when strains carry an intact *cag* pathogenicity island (*cag* PAI); a group of virulence genes specific to *H. pylori* (Sharma et al., 1998; Ando et al., 1996). Clinical biopsy-based studies complement these findings. For example, in antral mucosa of infected patients, IL-8 activity measured in biopsy homogenates is significantly higher than in uninfected controls and correlates with both bacterial density and histological activity scores (Gionchetti et al., 1994). Quantitative analyses of antral tissue homogenates demonstrate concentrations of IL-8 and IL-6 were higher in *H. pylori* positive dyspeptic patients compared with negative subjects, reinforcing the view that chemokine production is tightly linked to the intensity of mucosal inflammation (Gionchetti et al., 1994). Immunohistochemical localization further shows IL-8 protein within surface epithelial cells and CD68 positive macrophages in the lamina propria, establishing a direct connection between epithelial sensing and recruitment of inflammatory cells (Crabtree et al., 1994).

Recent studies further indicate that IL-8 findings are dependent on the biological compartment and analytical method, because circulating and gastric-tissue measurements do not necessarily demonstrate the same directional change (Saaed et al., 2021; Yang et al., 2024).

These chemokine gradients rapidly attract neutrophils and monocytes into the lamina propria and surface epithelium, where they release reactive oxygen and nitrogen species (ROS and NOS), myeloperoxidase (MPO), elastase (ELA), and matrix metalloproteinases (MMP) that contribute to epithelial damage and glandular distortion (Dixon et al., 2019; Crabtree et al., 1994). At the same time, epithelial cells upregulate antimicrobial peptides including human β defensin 2 and 3(hβD-2 and hβD-3). However, the *H. pylori* effector CagA can suppress expression of human β defensin 3 by interfering with epidermal growth factor receptor dependent signaling, thereby weakening a key epithelial barrier defense despite ongoing inflammation (Bauer et al., 2012).

Several bacterial traits blunt the effectiveness of this innate storm. Urease mediated buffering of gastric acid reduces mucus viscosity and permits bacteria to reside in a narrow zone within the mucus where pH and oxidative stress are relatively less hostile (Karkhah et al., 2024b). Helical cell morphology and flagella driven motility facilitate rapid movement within this layer, allowing bacteria to escape focal areas of intense inflammation and maintain partial physical separation from infiltrating neutrophils (Karkhah et al., 2024b). The net result is a mucosa with markedly increased pro inflammatory mediator levels and leukocyte influx, but only modest reduction in bacterial load, setting the stage for the later adaptive phases of the immunokinetic profile (Dixon et al., 2019; Karkhah et al., 2024b).

Despite broad agreement that IL-8-driven neutrophil recruitment is a prominent feature of *H. pylori* gastritis, the magnitude and functional consequences of this response remain heterogeneous across studies. Recent findings also indicate that circulating and gastric tissue IL-8 measurements may not show identical directional changes, emphasizing the importance of biological compartment and analytical methodology. Moreover, intense neutrophilic inflammation does not necessarily translate into bacterial clearance, suggesting that neutrophils may simultaneously restrict bacterial expansion and contribute to tissue injury without achieving sterilizing immunity. Longitudinal studies integrating bacterial burden, neutrophil phenotype, cytokine profiles, and histopathological progression are needed to determine when neutrophilic responses shift from protective to predominantly pathogenic states (Saaed et al., 2021; Yang et al., 2024).

### 4.2 Th1 and Th17 polarization and cytokine cascades

As infection progresses beyond the first days, antigen bearing DCs in the gastric lamina propria sample *H. pylori* and migrate to draining lymphoid tissues. Human primary gastric DCs exposed to *H. pylori* upregulate costimulatory molecules and secrete IL-12, driving differentiation of Th1 cells that produce IFN-γ and support macrophage activation (Bimczok et al., 2010). Gastric DCs from infected individuals are particularly efficient at inducing IFN-γ producing CD4 T cells, underscoring their central role in shaping the adaptive response (Bimczok et al., 2010). Histologic and functional studies converge on the conclusion that *H. pylori*-associated gastritis is dominated by Th1 cytokines (Dixon et al., 2019). Increased expression of IFN-γ, TNFα, and IL-2 is observed in infected gastric mucosa and correlates with mononuclear and polymorphonuclear cell infiltration. More recent human gastric-mucosal studies have similarly reported increased IFN-γ expression and greater infiltration by IFN-γ-producing T cells (Bimczok et al., 2010; Dixon et al., 2019;, Kong et al., 2020). More pronounced Th1 skewing is typically seen in patients with peptic ulcer disease compared with those who have non ulcer dyspepsia, in line with the concept that an exuberant Th1 response contributes to tissue injury (Dixon et al., 2019).

In parallel, a substantial Th17 component develops. Th17 cells secreting IL-17A, IL-17F, IL-21, and IL-22 accumulate in the gastric mucosa and in peripheral blood of infected patients ( (Dixon et al., 2019; Karkhah et al., 2024b). Experimental work indicates that *H. pylori* stimulates macrophages and DC to produce IL-23, IL-6, and chemokines such as CCL20 in an NF kappa B (NDKB) and MyD88 dependent manner, promoting Th17 differentiation (Liu et al., 2016). Human studies generally report increased IL-17A production by gastric T cells from infected individuals, especially those with intestinal metaplasia or dysplasia, whereas IL-4 production remains minimal, highlighting the predominantly pro inflammatory profile of these cells (Della Bella et al., 2023). Recent human studies generally report increased IL-17A-related measures in gastric tissue and peripheral blood, particularly in chronic gastritis and premalignant lesions, although the magnitude of these responses varies according to disease stage, biological specimen, and analytical methodology (Della Bella et al., 2023; Zhu et al., 2025). Th17 derived cytokines reinforce neutrophil recruitment and activation and enhance epithelial secretion of chemokines and antimicrobial peptides, thereby contributing to bacterial control but also amplifying mucosal damage. Within individual studies, gastric IL-17 levels have been reported to correlates positively with inflammatory scores and neutrophil counts and inversely with bacterial density, suggesting that Th17 responses participate in limiting bacterial expansion even though eradication is rarely achieved (61-62). Collectively, the evolving Th1 and Th17 cascades generate a sustained environment rich in IFN-γ, IL-17, IL-21, and IL-22 that both constrains and injures the gastric mucosa (Dixon et al., 2019; Liu et al., 2016).

Although the available evidence consistently supports Th1/Th17 polarization during *H. pylori* infection, the relative contribution of these responses to protection versus pathology remains unresolved. Increased IFN-γ and IL-17A may contribute to bacterial restriction, yet neither response is sufficient for eradication and both can amplify mucosal injury. Furthermore, reported cytokine responses vary with disease stage, bacterial virulence profile, host background, biological compartment, and analytical method. Thus, increased Th1/Th17 activity should not be interpreted uniformly as either protective or pathogenic. Longitudinal studies are needed to determine whether specific cytokine trajectories, rather than single measurements, predict persistence, ulceration, or progression toward premalignant disease.

However, *H. pylori* simultaneously induces counter regulatory signals. Both *in vitro* and *in vivo* data show that infected antigen presenting cells (APCs) can produce IL-10 and other regulatory cytokines, promoting a shift towards Tregs responses and dampening full effector expansion (Liu et al., 2016). This dynamic interplay between pro inflammatory and regulatory pathways is central to the immunokinetic trajectory and underlies the failure to reach sterilizing immunity.

### 4.3 Regulatory T cell dynamics and immune exhaustion

Tregs are a defining feature of chronic *H. pylori* infection. Gastric biopsies from infected patients contain increased numbers of CD4, CD25, high FOXP3 expressing Treg cells compared with uninfected controls, in both the antrum and duodenum (Lundgren et al., 2005). These mucosal FOXP3 positive cells express high levels of CTLA-4 and possess potent suppressive activity, inhibiting *H. pylori* specific T cell proliferation and IFN-γ production (Lundgren et al., 2005). Quantitative analyses indicate that Tregs can represent a substantial fraction of the CD4 compartment in infected mucosa, and their numbers are further increased in gastric adenocarcinoma tissue relative to adjacent non tumorous areas (Lundgren et al., 2005; Cheng et al., 2012; Ma et al., 2026).

Additional clinical series confirm that Foxp3 positive Tregs accumulate across the spectrum of *H. pylori* associated disease (Cheng et al., 2012; Urakawa et al., 2021). Patients with chronic gastritis, peptic ulcer, and gastric adenocarcinoma exhibit higher numbers of Foxp3 positive cells than uninfected individuals, and Tregs density often correlates positively with the grade of chronic and atrophic gastritis (Cheng et al., 2012). A focused review of human data emphasizes that these cells likely suppress excessive effector responses and thereby facilitate persistent colonization (65). Importantly, eradication therapy leads to significant reductions in mucosal *FOXP3* and *IL-10* mRNA expression, supporting a direct link between bacterial presence and local Tregs induction (Lundgren et al., 2005; Romero-Adrian et al., 2010).

At the molecular level, Tregs exert their suppressive activity through cytokines such as IL-10 and TGFβ1 as well as through contact dependent mechanisms involving CTLA 4. These signals reduce antigen presentation capacity, inhibit costimulatory molecule expression on DCs, and directly limit proliferation and cytokine production by effector Th1 and Th17 cells (Ando et al., 1996; Karkhah et al., 2024b). Over time, chronic antigen exposure in the inflamed gastric mucosa is associated with upregulation of inhibitory receptors such as PD-1 on T cells and increased PD-L1 expression on gastric epithelial cells and APCs (Araújo and Belo, 2016; Ohkusa et al., 2009). Engagement of the programmed cell death protein 1 (PD-1) and programmed death-ligand 1 PD-L1 axis transduces inhibitory signals that decrease T cell proliferation, cytokine production, and cytotoxicity, contributing to a state of functional exhaustion characteristic of chronic infections and tumors (Romero-Adrian et al., 2010; Ohkusa et al., 2009).

In *H. pylori* infection, PD-L1 expression is increased on gastric epithelial cells from infected subjects, and interaction with PD-1 positive T cells promotes T cell apoptosis and immune tolerance (Ohkusa et al., 2009). Reviews of the PD-1 PD-L1 checkpoint in this context argue that activation of this pathway is one mechanism by which *H. pylori* maintains long term persistence and, in some patients, contributes to immune escape during gastric carcinogenesis (Araújo and Belo, 2016). Together, expansion of Tregs, production of suppressive cytokines, and engagement of immune checkpoints generate a regulatory network that constrains effector immunity. Histologically, inflammation remains evident, but effector T cells become progressively less capable of clearing bacteria, and many display features of exhaustion. coinciding with the immunokinetic dynamics of *H. pylori* infection (early IL8, Th1/Th17 peaks, sustained systemic IgG, lower mucosal IgA, and bacterial load decline as IgA rises) as shown in **Table 3**, **Figures 2**, **3**.

**Figure 2:**
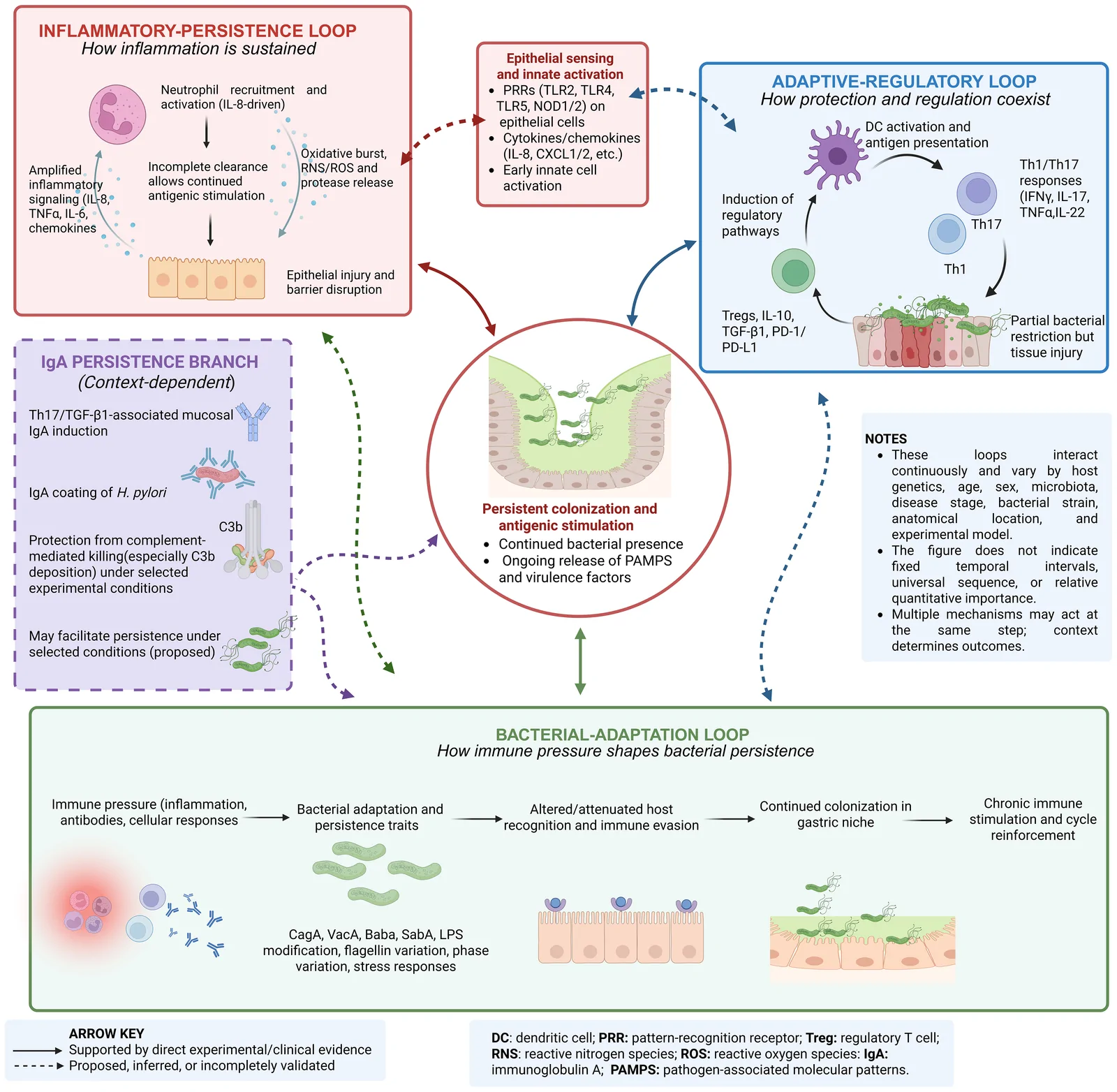
Evidence-informed feedback loops within the conceptual immunokinetic framework of *Helicobacter pylori* infection. The inflammatory-persistence loop links epithelial sensing and cytokine release with neutrophil recruitment, oxidative and nitrosative injury, incomplete bacterial clearance, and continued antigenic stimulation. The adaptive-regulatory loop links dendritic-cell activation, Th1/Th17 responses, partial bacterial restriction, tissue injury, regulatory T-cell activity, regulatory cytokines, immune-checkpoint signaling, and reduced effector clearance. The bacterial-adaptation loop illustrates how host immune pressure may interact with bacterial persistence traits, altered host recognition, continued colonization, and chronic immune stimulation. The context-dependent IgA branch illustrates experimental evidence that IgA coating may protect *H. pylori* from complement-mediated killing under selected conditions. Solid arrows represent interactions supported by direct experimental or clinical evidence, whereas dashed arrows represent proposed, inferred, or incompletely validated relationships. The diagram does not indicate fixed temporal intervals, a universal sequence, or relative quantitative importance. Representative supporting studies are cited in Section 4.4.

**Figure 3:**
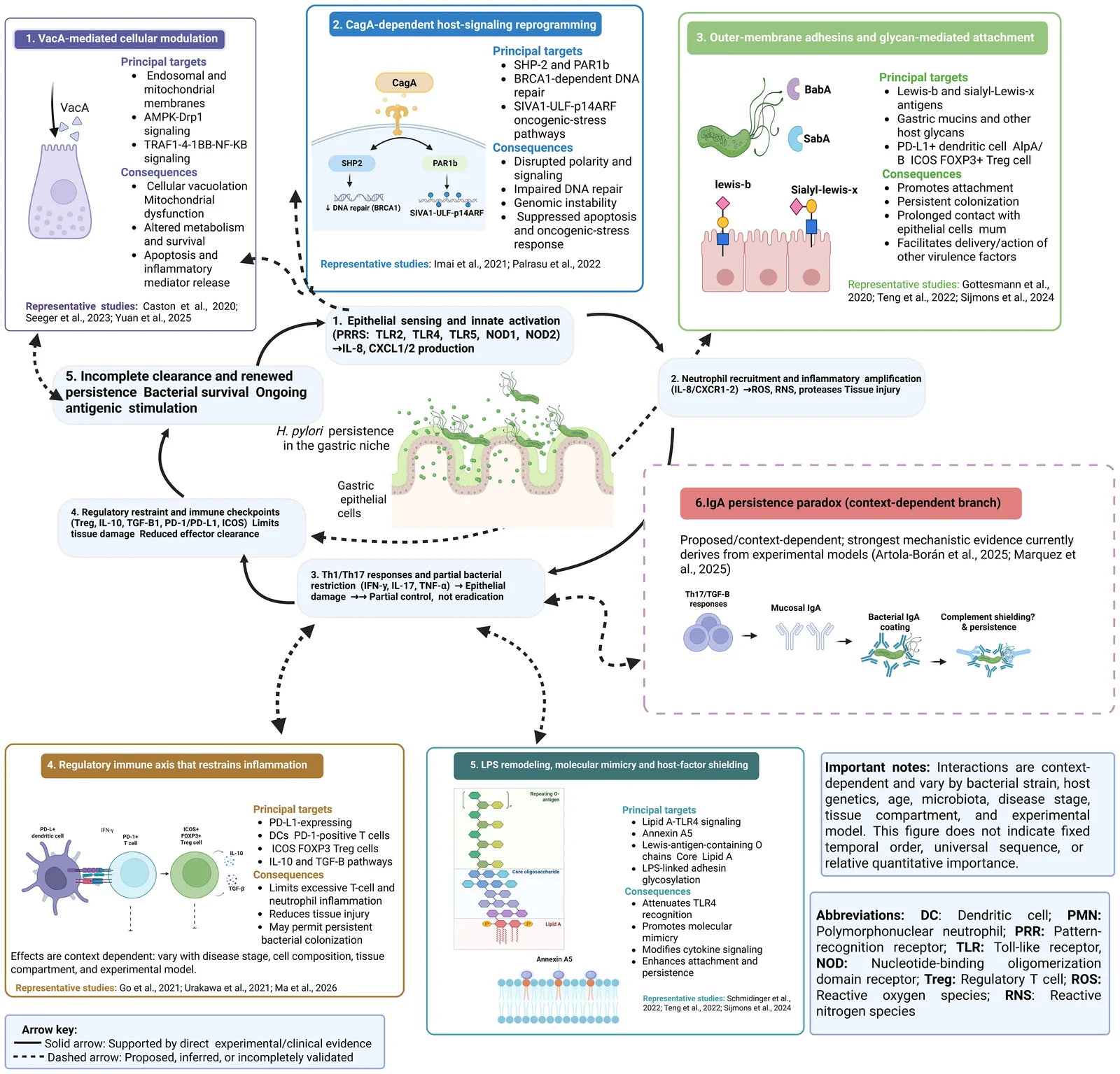
Context-dependent persistence mechanisms during *H. pylori* infection. Major bacterial and host-mediated mechanisms contributing to persistent *H. pylori* colonization are illustrated without assigning numerical or qualitative rankings. VacA modulates epithelial metabolism, mitochondrial function, and cell survival; CagA reprograms host signaling and DNA-repair pathways; outer-membrane adhesins promote sustained attachment to gastric epithelial cells; regulatory immune pathways restrain inflammation while potentially facilitating bacterial persistence; and LPS remodeling contributes to reduced innate immune recognition, molecular mimicry, and host-factor interactions. The magnitude and biological consequences of these mechanisms vary according to bacterial strain, virulence-factor genotype, host characteristics, disease stage, cellular context, and experimental model.

Quantitative cytokine measurements in *H. pylori* infection should be interpreted in light of substantial biological and methodological heterogeneity. Reported concentrations may vary according to age, sex, ethnicity, host genetic background, infecting bacterial strain and virulence profile, disease stage and histopathological severity, and the biological compartment examined, including gastric biopsy homogenates, mucosal samples, plasma, or serum. Additional variability may arise from specimen collection and processing, assay platform, calibration standards, detection limits, normalization procedures, and individual laboratory protocols. Consequently, the values summarized in **Table 3** represent study-specific ranges rather than standardized reference intervals. Absolute cytokine concentrations should therefore be interpreted cautiously and primarily reflect trends rather than universal diagnostic thresholds.

*The direction of change pertains to the comparator and the assessed endpoint utilized in each referenced study. Variable” indicates discrepancies based on the biological compartment, comparator group, disease stage, bacterial genotype, patient attributes, specimen-processing technique, or analytical approach. They are provided to illustrate reported trends and should not be interpreted as standardized reference intervals, universal diagnostic thresholds, or directly comparable values across studies.

These observations highlight an unresolved trade-off in *H. pylori* immunity: regulatory responses that protect the gastric mucosa from excessive inflammatory injury may simultaneously prevent sterilizing immunity. It therefore remains unclear whether therapeutic attenuation of Treg-, IL-10-, or checkpoint-mediated suppression would improve bacterial control without causing unacceptable tissue damage. Future studies should distinguish regulatory mechanisms that primarily maintain physiological mucosal tolerance from those specifically exploited by *H. pylori* to establish persistence.

### 4.4 Integrated immunokinetic framework

When the above-mentioned components are integrated, a characteristic immunokinetic profile emerges. Early in infection of infection, epithelial and innate cells detect *H. pylori* via PRRs and rapidly induce IL-8, IL-1β, TNFα, IL-6, and antimicrobial peptides, leading to neutrophil and monocyte recruitment (Cheok et al., 2022; Sharma et al., 1998; Bauer et al., 2012). Available evidence suggests that innate, adaptive, and regulatory responses develop as partially overlapping phases rather than as a universally fixed sequence. Following early epithelial and myeloid-cell activation, Th1- and Th17-associated responses generally become more prominent, although their timing and magnitude vary according to the host, bacterial strain, anatomical compartment, and disease context. Early in infection, antigen presentation by gastric DCs primes Th1- and Th17-associated responses, generally accompanied by increased IFN-γ-, IL-17-, and IL-21-related measures in gastric mucosa or peripheral blood. However, the timing and magnitude of these changes vary across biological compartments and analytical methods (Kong et al., 2020; Della Bella et al., 2023; **Table 3**). During this period, bacterial numbers initially decline but then stabilize at a new steady state, reflecting partial immune control rather than eradication (Dixon et al., 2019). Regulatory circuits progressively intensify. Mucosal FOXP3 positive Tregs expand, and their cytokine products IL-10 and TGFβ1 accumulate in the tissue. At the same time, expression of PD-L1 on gastric epithelial cells and APCs and PD-1 on T cells increases, promoting T cell apoptosis and functional exhaustion (Lundgren et al., 2005; Ohkusa et al., 2009). This regulatory dominance restrains excessive tissue destruction but also prevents the immune system from eliminating the bacteria, locking the host into a chronic inflammatory equilibrium.

Collectively, these observations support the concept that *H. pylori* persistence is not merely the consequence of immune evasion alone, but rather the outcome of a dynamic immunokinetic equilibrium in which inflammatory activation, regulatory suppression, bacterial adaptation, and epithelial remodeling continuously counterbalance one another. This framework may help explain the remarkable clinical heterogeneity observed between asymptomatic colonization, ulcerative disease, and gastric carcinogenesis.

Humoral immunity follows a somewhat slower trajectory. *H. pylori* specific IgG appears in serum and IgA in gastric secretions and mucosal homogenates after colonization, indicating active B cell responses at both systemic and mucosal levels (Bogstedt et al., 1996). However, studies in individuals with IgA deficiency and in vaccine models challenge the notion that secretory IgA is necessarily protective. Patients with selective IgA deficiency do not show a lower prevalence of *H. pylori* infection compared with controls, suggesting that secretory IgA alone is not sufficient for clearance (Bogstedt et al., 1996). In mouse models and experimental vaccination, strong Helicobacter specific IgA responses can \ impair protective immunity, in part by altering antigen presentation and effector cell access to bacteria (Blanchard et al., 2005). Recent work in mice and human gastritis has extended this view by showing that *H. pylori* triggers robust local IgA production, and that IgA coated bacteria can persist in the mucus; Emerging evidence suggests that under specific conditions IgA-coated bacteria may exhibit enhanced persistence (Artola-Borán et al., 2025), indicating that IgA-mediated immunity is not uniformly protective.

Based on the evidence summarized above, we organize the reported interactions into three interconnected feedback loops within the conceptual immunokinetic framework (**Figure 2**). First, the inflammatory-persistence loop links epithelial pattern-recognition receptor activation and cytokine or chemokine release with IL-8-associated neutrophil recruitment, oxidative and nitrosative stress, epithelial injury, and incomplete bacterial clearance. Continued colonization may then sustain antigenic stimulation and recurrent inflammatory signaling (Jang et al., 2020; Cheok et al., 2022; Yang et al., 2024; Karkhah et al., 2024b). Second, the adaptive-regulatory loop links dendritic-cell activation and Th1/Th17 responses with partial bacterial restriction and tissue injury, followed by Treg-, IL-10-, TGF-β1-, and PD-1/PD-L1-mediated restraint. This regulatory response may limit excessive immunopathology while simultaneously reducing effective bacterial clearance (Bimczok et al., 2010; Lundgren et al., 2005; Ohkusa et al., 2009; Go et al., 2021; Della Bella et al., 2023). Third, the bacterial-adaptation loop links host immune pressure with context-dependent persistence traits involving CagA and VacA (Caston et al., 2020; Imai et al., 2021), adhesin and glycan interactions (Teng et al., 2022; Sijmons et al., 2024), and LPS- or flagellin-related modulation of host recognition (Neuper et al., 2020; Schmidinger et al., 2022). Together, these processes may support continued colonization and renewed immune stimulation.

The individual components of these loops are supported to different degrees by experimental and clinical evidence; however, their integration into closed feedback cycles, their temporal ordering, and their relative importance represent hypothesis-generating interpretations that require prospective longitudinal validation.

A related context-dependent branch may involve Th17- and TGF-β-associated IgA induction, bacterial IgA coating, and protection from complement-mediated clearance, although the extent to which this mechanism contributes to persistence in humans remains uncertain (Artola-Borán et al., 2025).

### 4.5 Relationship to existing models and intended contribution

Previous models of *H. pylori* pathogenesis have primarily focused on individual components of the host-pathogen interaction, including innate immune activation, Th1/Th17 polarization, regulatory T-cell responses, bacterial virulence factors, or gastric carcinogenesis. While the present framework does not replace these models. Rather, it organizes established components within a temporal and integrative perspective and highlights relationships that may warrant prospective investigation. The immunokinetic framework proposed in this review differs from conventional models by integrating multiple dimensions of host-pathogen interaction into a unified temporal perspective. Rather than viewing *H. pylori* infection as a static chronic inflammatory state, this framework conceptualizes infection as a continuously evolving process characterized by sequential and overlapping phases of immune activation, immune regulation, bacterial adaptation, tissue remodeling, and clinical progression.

A central feature of this framework is the incorporation of cytokine kinetics. Early innate responses dominated by IL-8, IL-1β, and TNF-α are followed by the emergence of Th1- and Th17-associated cytokines such as IFN-γ and IL-17, which appears to overlap with regulatory mediators including IL-10 and TGF-β. These temporal cytokine patterns are considered alongside changes in bacterial burden and immune-cell composition, providing a more dynamic representation of disease progression. The framework additionally integrates bacterial adaptation mechanisms, including ***v****acA*-mediated immunomodulation, ***c****agA*-dependent signaling reprogramming, adhesin switching, and attenuation of innate immune recognition through modified LPS and flagellin structures. These bacterial strategies are not viewed as isolated virulence traits but rather as adaptive responses that continuously interact with evolving host immunity.

Importantly, the model links immune dynamics directly to clinical outcomes. Variations in the balance between inflammatory and regulatory responses may help explain why some individuals remain asymptomatic, whereas others progress toward peptic ulcer disease, atrophic gastritis, intestinal metaplasia, or gastric adenocarcinoma. By connecting temporal immune responses, cytokine kinetics, bacterial adaptation, and pathological outcomes within a single conceptual framework, the immunokinetic framework provides a testable interpretation of how temporal immune responses, bacterial adaptation, and pathological outcomes may be interconnected. This integrative perspective may also facilitate the development of biomarker-guided diagnostics, personalized risk stratification strategies, and targeted immunomodulatory interventions, thereby providing a translational framework for future research and clinical applications.

## 5 Humoral immunity: IgG versus IgA dynamics

### 5.1 Serological IgG: utility and limitations

Systemic humoral responses to *H. pylori* are dominated by IgG directed against multiple bacterial antigens, including urease, CagA, VacA and several outer membrane proteins. Virtually all chronically infected adults develop circulating IgG that is readily detected by enzyme linked immunosorbent assays and immunoblots, and titers generally rise with bacterial load and duration of colonization (Blanchard et al., 1999). In early human studies, patients with active infection showed broad IgG reactivity to *H. pylori* proteins, whereas an individual who spontaneously cleared infection displayed marked changes in the antibody profile, underlining the link between persistent antigenic stimulation and sustained IgG production (Blanchard et al., 1999).

In clinical practice, IgG based assays became the mainstay of noninvasive diagnosis for many years. Evaluations against culture, histology and urea breath test standards show that well validated IgG ELISA kits usually achieve sensitivities in the range of eighty to ninety percent and specificities around eighty percent, particularly in high prevalence settings (Saadi and Saeed, 2018; Peterson, 2000). These characteristics make IgG serology particularly well suited for epidemiologic work and for first line case finding where endoscopy, urea breath testing or monoclonal stool antigen assays are not easily available (Malfertheiner et al., 2022; Cheng et al., 2012; Saadi and Saeed, 2018; Peterson, 2000).

The relative dominance of IgG over other isotypes is illustrated by detailed serological studies. In a hospital cohort from Duhok in the Kurdistan region of Iraq, ninety percent of whom underwent endoscopy for dyspepsia, ELISA seroprevalent testing showed IgG positivity in about seventy one percent of patients, whereas IgA and IgM were detected in only about thirty and six percent respectively when culture was used as the reference standard (Saadi and Saeed, 2018). The authors also noted that IgG positivity increased with age, consistent with cumulative acquisition of infection over time (Saadi and Saeed, 2018).

Comparable patterns emerge from sudanese cohorts. In a cross-sectional study from Tamboul Hospital in Gezira State, Sudan, symptomatic and asymptomatic outpatients were screened with immunochromatographic kits for serum antibodies and stool antigen. Serum antibody positivity reached sixty percent in symptomatic and fifty percent in asymptomatic participants, while stool antigen positivity, which reflects current colonization, was present in seventy and thirty six percent respectively (Abd Alla, 2020). Taken together, these data highlight that systemic IgG responses are common even in apparently healthy carriers and that seroprevalence substantially exceeds the proportion with active symptomatic disease (Blanchard et al., 1999; Saadi and Saeed, 2018; Abd Alla, 2020).

From a kinetic standpoint, serum IgG declines slowly after successful eradication. Consensus reports and practice guidelines emphasize that antibody titers may remain positive for many months and often for several years after treatment, so that serology is a poor test of eradication at the individual level (Malfertheiner et al., 2022; Abd Alla, 2020). A guideline paper from the late 1990s has already pointed out that high IgG levels remain for “variable and extended periods” after therapy and that seroconversion more than a year after treatment is a reliable but delayed marker of cure (Peterson, 2000). In the Maastricht VI Florence expert guidelines, recommend against relying on IgG serology to confirm eradication, favoring functional assays instead (Malfertheiner et al., 2022). Thus, persistent IgG is best interpreted as an imprint of lifetime exposure rather than as a real time marker of mucosal protection or ongoing colonization (Malfertheiner et al., 2022; Blanchard et al., 1999; Peterson, 2000).

### 5.2 Mucosal IgA and protective immunity

In contrast to IgG, secretory IgA is the dominant immunoglobulin at mucosal surfaces. In the gastric environment, polymeric IgA transported across the epithelium via the polymeric immunoglobulin receptor can bind bacterial surface structures, interfere with adhesin receptor interactions and neutralize soluble toxins, thereby contributing to immune exclusion of *H. pylori* from the epithelial surface (Blanchard et al., 1999; Czinn and Blanchard, 2011; Ding et al., 2022). Human biopsy material demonstrates marked expansion of IgA producing plasma cells in the gastric lamina propria of infected individuals, and *H. pylori* specific IgA is readily detected in gastric juice and mucosal washes (Blanchard et al., 1999; Czinn and Blanchard, 2011).

Nevertheless, natural infection data suggest that mucosal IgA is neither strictly necessary nor sufficient for protection. In patients with selective IgA deficiency, the prevalence of *H. pylori* infection and the severity of associated gastritis are similar to those in immunocompetent controls, despite the absence of secretory IgA and dependence on IgG and IgM for humoral defense (Bogstedt et al., 1997). In the previous cohort, IgA deficiency did not predispose to uncontrolled colonization or more severe peptic disease, indicating that other immune effectors can compensate for the lack of IgA in the gastric niche (Bogstedt et al., 1997).

Recent experimental work has further complicated the simple view of IgA as purely protective. Artola Borán et al. examined IgA responses in mouse models and in human gastritis tissue and found that *H. pylori* induces a strong Th17 dependent local IgA response that results in dense IgA coating of bacteria (Artola-Borán et al., 2025). Class switching to IgA required Peyer patches, T cells and eosinophil derived TGFβ1, and depended on a functional bacterial type four secretion system that also drove Th17 differentiation and expression of the polymeric immunoglobulin receptor (Artola-Borán et al., 2025). Strikingly, however, mice lacking IgA controlled *H. pylori* more efficiently than wild type animals, with lower bacterial loads in gastric tissue (Artola-Borán et al., 2025). Mechanistic experiments indicated that IgA could protect *H. pylori* from complement mediated killing, and that elimination of complement component C3 abolished the advantage of IgA deficiency (Artola-Borán et al., 2025). These findings suggest that high affinity IgA may sometimes serve as an immune shield that favors persistence of this particular pathogen rather than its elimination.

These findings challenge the traditional assumption that stronger mucosal antibody responses necessarily correlate with improved host protection. Instead, they suggest that the functional consequences of IgA responses in *H. pylori* infection may depend on antibody specificity, glycosylation patterns, complement interactions, and the surrounding inflammatory microenvironment. This emerging “IgA paradox” highlights the complexity of mucosal immunity in chronic gastric infection and has important implications for vaccine design strategies that aim to induce durable secretory IgA responses.

These observations align with broader work on IgA biology. Reviews of IgA glycosylation show that structural variants of IgA can tune its interaction with Fc alpha receptors, thereby modulating pro inflammatory or anti-inflammatory signaling on myeloid cells (Ding et al., 2022). In the gastric mucosa, altered glycosylation of *H. pylori* specific IgA could potentially influence immune complex formation, clearance and the balance between protective neutralization and chronic inflammatory stimulation (Ding et al., 2022; Fuchs et al., 2024). Mucosal IgA in *H. pylori* infection emerges as a context-dependent effector. On one hand it contributes to neutralization of bacterial products, limits epithelial damage and may reduce colonization at high local concentrations. On the other hand, specific patterns of IgA response and IgA coating of bacteria can protect *H. pylori* from complement and phagocyte mediated killing, supporting long term colonization and the immunokinetic paradox of strong inflammation without sterilizing immunity (Artola-Borán et al., 2025; Bogstedt et al., 1997; Fuchs et al., 2024).

The apparently opposing effects of IgA challenge the assumption that the magnitude of a mucosal antibody response directly predicts protection. A more relevant determinant may be antibody quality including specificity, affinity, glycosylation, anatomical localization, and interaction with complement rather than total IgA abundance alone. Whether distinct IgA signatures differentiate protective from persistence-promoting responses remains an important unresolved question with direct implications for mucosal vaccine design.

### 5.3 Diagnostic and clinical applications

Understanding IgG and IgA dynamics has direct diagnostic implications. Serum IgG remains a pragmatic tool for epidemiologic surveys and initial risk stratification in resource constrained settings. Its advantages include wide availability, low cost and the ability to capture both current and past infection at the population level (Malfertheiner et al., 2022; Saadi and Saeed, 2018; Peterson, 2000). However, because titers decline only slowly after eradication, serology cannot reliably distinguish active infection from remote exposure in individual patients and should not be used as a standalone test of cure (Malfertheiner et al., 2022; Peterson, 2000).

The diagnostic performance of IgA based assays is more limited. In the Kurdistan evaluation that used culture as the reference standard, IgG ELISA identified about seventy one percent of patients as positive, whereas IgA identified about thirty percent, and IgA based testing showed lower sensitivity and overall accuracy than IgG (Saadi and Saeed, 2018). Other comparative studies and guideline summaries report IgA sensitivities typically in the sixty to seventy percent range and emphasize that IgA serology adds little when high quality IgG assays and functional tests are available (Malfertheiner et al., 2022; 74). For this reason, major consensus documents do not recommend IgA alone for routine diagnosis, although combined IgG and IgA measurements can be informative in research settings where the aim is to characterize patterns of humoral response or to distinguish recent from distant exposure in sero-epidemiological cohorts (Malfertheiner et al., 2022; Fuchs et al., 2024; Santacroce et al., 2025; Márquez-Moñino et al., 2025).

For confirmation of active infection and of eradication after therapy, functional non serologic tests are now preferred. The Maastricht VI Florence report and earlier practice guidelines place urea breath tests and monoclonal stool antigen assays at the center of the diagnostic algorithm, because they directly detect ongoing colonization and achieve sensitivities and specificities above ninety percent when used appropriately with respect to acid suppressive and antimicrobial drugs (46, 55). Serology is mainly reserved for circumstances where urea breath testing and stool antigen assays are unavailable or contra indicated, or where pretest probability is high and epidemiologic rather than individual level precision is the primary concern (Malfertheiner et al., 2022; Peterson, 2000).

At a conceptual level, the contrasting behavior of IgG and IgA illustrates the broader immunokinetic theme of *H. pylori* infection. Persistent systemic IgG provides a durable serological footprint of exposure and is valuable for mapping the burden of infection, while mucosal IgA participates in complex networks that can both constrain and sustain chronic colonization. Integrating these humoral markers with cellular immune profiles and with clinical outcomes adds an important layer of nuance to our understanding of why *H. pylori* infection so often persists despite vigorous immune activation (Blanchard et al., 1999; Bogstedt et al., 1997; Ding et al., 2022; Santacroce et al., 2025).

## 6 Immune evasion mechanisms

### 6.1 VacA and CagA: key virulence factors

Vacuolating cytotoxin A (Vac A) is secreted as a protoxin that is processed to form oligomeric channels able to insert into endosomal and mitochondrial membranes (Seeger et al., 2023). In epithelial cells this leads to impaired endosomal acidification, cytoplasmic vacuolation and mitochondrial damage with cytochrome c release and apoptosis, illustrating how a single toxin can simultaneously affect membrane trafficking and cell survival pathways (Cover and Blanke, 2005; Isomoto et al, 2010). VacA also modifies host signaling by engaging several surface receptors and perturbing autophagy and stress responses, effects that differ between epithelial cells, lymphocytes and APCs (Backert and Tegtmeyer, 2010).

In the immune compartment, VacA directly interferes with T cell activation. It blocks calcium dependent signaling downstream of the T cell receptor and IL-2 receptor, inhibits nuclear factor of activated T cells translocation and reduces IL-2 driven proliferation of human T cells *in vitro* (Cover and Blanke, 2005). VacA further impairs major histocompatibility complex class two (MHC) antigen processing and promotes a semi mature dendritic cell phenotype with high antigen presentation but low costimulatory capacity, biasing subsequent T cell priming away from full effector differentiation (Higashi et al., 2004). These combined actions establish VacA as a central mediator of functional immunosuppression during infection (Yamaoka, 2010; Higashi et al., 2004).

Cytotoxin associated protein (CagA) A is translocated into gastric epithelial cells through the *cag* pathogenicity island encoded type four secretion system (Higashi et al., 2004). Following tyrosine phosphorylation on EPIYA motifs, CagA binds SHP2 and induces its aberrant activation, which drives persistent extracellular signal regulated kinase signaling, cytoskeletal rearrangements and the elongated hummingbird morphology (Higashi et al., 2004; Mohammadzadeh et al., 2023). Structural and functional work has shown that CagA also interacts with polarity regulators and junctional proteins, so that epithelial polarity, tight junctions and contact inhibition are all progressively disrupted in CagA expressing cells (Hatakeyama, 2017; Higashi et al., 2002). In long term models, these alterations cooperate with inflammation induced damage to promote genomic instability and a cancer associated transcriptional programmed, validating CagA as a bacterial oncoprotein (Higashi et al., 2002; Palrasu et al., 2022). Besides ***c****agA* -driven signaling reprogramming, the adhesion mediated by BabA/SabA to Lewis (Le) antigens promotes epithelial colonization and supports prolonged host–pathogen interaction. BabA and SabA contribute to gastric attachment by recognizing different host glycan structures. However, their relative roles vary according to bacterial strain, phase-variable expression, host glycosylation patterns, anatomical site, and inflammatory stage. Adhesin functions are also partly redundant; therefore, universal percentage contributions or a fixed temporal predominance should not be assigned (Gottesmann et al., 2020; Teng et al., 2022; Sijmons et al., 2024, **Figure 3**).

### 6.2 Outer membrane adhesins and antigenic variation

Stable colonization requires firm attachment to gastric mucus and epithelial surfaces. The best characterized adhesins, BabA and SabA, bind fucosylated Le blood group antigens and sialylated Lewis antigens respectively, anchoring the bacterium to the epithelial glycocalyx. As inflammation remodels surface glycosylation from non sialylated to sialylated structures, expression of these adhesins is regulated by phase variation and gene conversion so that binding is maintained despite dynamic changes in host receptors (Pachathundikandi et al., 2023). Broader reviews emphasize that similar phase variable control is applied to many outer membrane proteins, generating heterogeneous subpopulations that differ in adhesin profiles and antigenic visibility, which reduces the effectiveness of antibody mediated clearance (Mohammadzadeh et al., 2023).

At the level of innate immune recognition, *H. pylori* modifies classical pathogen-related molecular patterns (PRMP). Its LPS contains under acylated and hypo-phosphorylated lipid A species that are weak agonists for TLR-4, shifting innate sensing toward other receptors and dampening pro inflammatory signaling that would otherwise promote rapid bacterial elimination (Gewirtz et al., 2004). In addition, core and O-chain structures often mimic host Le blood group antigens, which further reduces immune detection and facilitates coexistence within the inflamed mucosa (Gewirtz et al., 2004; Oertli et al., 2010).

Flagellin shows analogous adaptation. Mutations and deletions in the TLR-5 recognition motif allow the bacterium to retain motility while largely avoiding TLR-5 mediated NF kappa B activation, so that a major danger signal is effectively silenced (Neuper et al., 2020). Dendritic cell studies confirm that TLR-2, 4 and 10 rather than TLR-5 dominate the cytokine and chemokine response to *H. pylori*, consistent with masking or biochemical modification of the usual ligands for TLR-4 and 5 (Liu et al., 2023). Integrative analyses therefore describe a coordinated strategy in which adhesins secure the niche, while LPS and flagellin are remodeled to attenuate innate receptor engagement and favor chronic colonization (Karkhah et al, 2024a; Gewirtz et al., 2004).

### 6.3 Regulatory T cells induction

Chronic infection is characterized not only by persistent effector responses but also by prominent Treg cells accumulation. Gastric biopsies from infected patients contain increased numbers of CD4 positive FOXP3 positive Tregs compared with uninfected controls, and these cells correlate with both bacterial density and the severity of chronic gastritis and neoplastic changes (Mitchell et al., 2012). Functional assays indicate that they suppress proliferation and cytokine production by Helicobacter specific effector T cells, thereby limiting tissue damage but also restricting bacterial clearance (Mitchell et al., 2012).

A mechanistic link between innate sensing and regulatory expansion has been demonstrated using human DCs and T cell cocultures. *H. pylori* stimulated DCs produce IL-1 β and drive *in situ* proliferation of Tregs; the fraction of cycling FOXP3 positive cells in gastric tissue is significantly higher in infected than uninfected mucosa, indicating local expansion at the site of infection (Gerhard and Rad, 2016). Blocking IL-1β pathways reduces this proliferation, supporting a model in which dendritic cell derived cytokines connect bacterial recognition to the regulatory arm of the response.

In addition to promoting Tregs expansion, *H. pylori* virulence factors such as VacA actively contribute to the reinforcement of a tolerogenic immune environment. In mouse models and human cell systems, VacA exposure maintains DCs in a semi mature state with altered cytokine secretion and reduced ability to support robust effector T helper responses (97). These VacA conditioned DCs preferentially induce Tregs that stably express FoxP3 and CD25 and exert strong suppressive activity. Infection with VacA deficient strains is associated with fewer Tregs, more intense Th1 and Th17 responses and lower bacterial colonization, whereas complementation with VacA restores the regulatory bias and persistence phenotype (Adamsson et al., 2017; D'Elios and Della Bella, 2024).

A limitation of studying these virulence determinants individually is that naturally infecting *H. pylori* strains carry different combinations and allelic variants of ***c****agA*, ***v****acA*, *babA*, *sabA*, and other immune-modulatory genes. Their biological effects are therefore unlikely to be simply additive or uniform across hosts. Furthermore, host genotype and the inflammatory microenvironment may modify the phenotypic consequences of the same bacterial virulence repertoire. Future studies should consequently move beyond single-factor associations toward integrated host–pathogen analyses that simultaneously characterize bacterial genotype, host immune state, and spatial tissue responses.

## 7 Clinical and translational insights

### 7.1 Cytokine profiles and pathology intensity

The associations summarized below should be interpreted as within-study trends because absolute cytokine concentrations are not directly comparable across populations, disease stages, biological specimens, and assay platforms.

At tissue level the severity of *H. pylori* associated pathology closely mirrors the local cytokine milieu. Biopsy-based studies show that gastric concentrations of IFN-γ and IL-17A rise in parallel with Sydney scores for neutrophilic activity- a histological grading system used to quantify the severity of gastric inflammation and tissue damage in biopsy specimen, including chronic inflammatory infiltrate and glandular damage, with the highest values in patients who have symptomatic gastritis or ulcer disease rather than uncomplicated colonization (D'Elios and Della Bella, 2024). Within individual cohorts, increased mucosal IFN-γ- and IL-17A-related measures have been associated with greater histological inflammatory activity and more advanced gastric lesions. However, these associations vary according to patient population, disease stage, specimen type, and analytical platform. IFN-γ and IL-17A should therefore be regarded as context-dependent indicators of inflammatory activity rather than universal diagnostic markers or quantitative cutoffs (Kong et al., 2020; Della Bella et al., 2023).

Counter regulatory cytokines are also strongly linked to histopathology. IL-10 is produced by the surface epithelium, lamina propria mononuclear cells and B cells in *H. pylori* associated gastritis, and its expression increases with the density of inflammatory infiltrate (Bodger et al., 2001). *In situ*, experimental neutralization of endogenous IL-10 in organ culture enhances the release of tumor necrosis factor alpha and IL-6, supporting the concept that IL-10 restrains local damage at the expense of more efficient bacterial clearance (Bodger et al., 2001). Clinically, patients who progress from non-ulcer dyspepsia to peptic ulcer disease tend to exhibit a higher ratio of proinflammatory mediators such as TNF-α and IL-1β to IL-10, together with biochemical indices of oxidative stress, consistent with a shift from controlled chronic inflammation toward a more destructive pattern of immune activation (Salavati et al., 2020; Zhu et al., 2025.

Collectively, these observations suggest that the translational value of cytokines may lie less in isolated absolute concentrations than in multidimensional patterns or longitudinal trajectories. A single cytokine is unlikely to discriminate reliably among uncomplicated colonization, active gastritis, ulcer disease, and premalignant progression across heterogeneous populations. Prospective studies using standardized sampling and analytical platforms are therefore required to determine whether composite cytokine signatures provide reproducible predictive information beyond conventional histopathology and clinical risk factors.

### 7.2 Metabolic and systemic associations

Beyond the gastric infection, chronic *H. pylori* infection appears to contribute to subtle systemic metabolic disturbance. A comprehensive meta-analysis pooling more than 200,000 participants found that *H. pylori* seropositivity was associated with an approximately 20% increase in odds of metabolic syndrome such as T2DM, and about 50% higher odds of insulin resistance, even after adjustment for classical cardiometabolic risk factors (Alzahrani et al., 2020). These data suggest that the chronic low grade inflammatory response to *H. pylori*, characterized by persistent production of cytokines such as tumor necrosis factor alpha and IL-6, may promote peripheral insulin resistance and dyslipidemia (Alzahrani et al., 2020; Azami et al., 2021).

Modulation of gastric hormones provides a complementary mechanism. In a physiological study that combined detailed endoscopic phenotyping with serial hormone measurements before and after eradication therapy, Francois and colleagues showed that successful clearance of *H. pylori* altered postprandial ghrelin and leptin dynamics without major changes in body mass index (Fragoulis et al., 2016). After eradication, the usual meal associated fall in ghrelin was attenuated while leptin concentrations increased, indicating that long standing infection can re set gut brain signaling pathways that regulate appetite and energy balance (Fragoulis et al., 2016).

Data from GCC populations underline the interaction between infection and metabolic traits in real world settings. A cross-sectional study from primary care centers in western Saudi Arabia reported *H. pylori* prevalence around twenty seven percent in both adults with type 2 diabetes and non-diabetic controls, but infection clustered with overweight and obesity, especially among non-diabetic individuals (Francois et al., 2011). Obese participants of either group were more likely to be *H. pylori* positive than lean counterparts, suggesting that shared behavioral and environmental exposures, as well as low grade inflammation, contribute to both infection and metabolic dysfunction (Francois et al., 2011). Taken together with global meta-analytic evidence, these regional observations support a model in which *H. pylori* acts as an additional inflammatory stressor that can modestly amplify cardiometabolic risk in genetically or environmentally susceptible Gulf populations (Alzahrani et al., 2020; Francois et al., 2011).

### 7.3 Therapeutic implications

From a therapeutic perspective the immunokinetic and systemic consequences of infection reinforce the rationale for early and effective eradication. The Maastricht VI and Florence consensus now explicitly classify *H. pylori* gastritis as an infectious disease for which all infected individuals should be offered treatment, irrespective of symptom burden, in order to prevent peptic ulcer disease and reduce gastric cancer risk (Malfertheiner et al., 2022). In regions where clarithromycin resistance remains low, 14-day proton pump inhibitor based triple therapy with amoxicillin and clarithromycin can still achieve per protocol eradication rates in the range of about seventy to eighty percent when adherence is good. However, in many areas rising resistance has shifted practice toward bismuth quadruple therapy (a proton pump inhibitor, bismuth, and two antibiotics) or non-bismuth concomitant regimens (a proton pump inhibitor plus three antibiotics without bismuth), which more consistently deliver eradication rates close to or above ninety percent and are therefore recommended as first line choices whenever local resistance data are unfavorable (Malfertheiner et al., 2022; Karkhah et al., 2024b; Fragoulis et al., 2016).

The immunological profile of chronic infection also points toward potential adjunctive and future strategies. An important future direction involves the integration of immunological biomarkers into risk-stratification models for *H. pylori*-associated disease. Dynamic cytokine signatures, including IFN-γ/IL-10 ratios, Th17-associated mediators, and immune checkpoint expression profiles, may eventually help distinguish patients likely to remain asymptomatic from those at higher risk of ulcerative or neoplastic progression. The potential value of incorporating immunokinetic parameters into clinical risk models requires evaluation in prospective, independently validated cohorts. Conceptually, Selective modulation of Th1, Th17, or regulatory pathways represents a hypothesis requiring preclinical evaluation because improved bacterial control could be accompanied by increased mucosal injury (Liu et al., 2023; D'Elios and Della Bella, 2024). Experience from other immune mediated diseases shows that selective blockade of IL-17 or IL-23 can dampen pathogenic inflammation without completely ablating host defense, raising the possibility that carefully targeted modulation of the Th17 axis might one day complement antimicrobial therapy in high-risk *H. pylori* associated disease (Wang et al., 2024).

At the same time, vaccine research increasingly uses mechanistic insight from T cell and antibody kinetics to design mucosal immunogens that elicit balanced helper responses and robust secretory IgA. Multicomponent vaccines incorporating antigens such as CagA, VacA and urease aim to generate antibodies that neutralize key virulence factors while promoting protective T helper 1 and T helper 17 responses at the gastric surface (Bodger et al., 2001). Parallel work on outer membrane vesicles and exosomes from infected cells has identified these vesicles as carriers of toxins, adhesins and immunomodulatory molecules, highlighting them as attractive candidates for novel vaccine platforms or monoclonal antibody targets that interfere with bacterial communication and persistence (Wang et al., 2024). In aggregate, these clinical and translational insights link the local cytokine landscape, systemic metabolic effects and evolving therapeutic strategies into a conceptual basis for future mechanistic and translational investigation. Nevertheless, translating these immunological principles into an effective human vaccine remains challenging. Natural infection already induces substantial Th1, Th17, and antibody responses without achieving clearance, indicating that simply increasing the magnitude of these responses may be insufficient and could potentially intensify gastric inflammation. An effective vaccine may therefore require a qualitatively distinct immune response that combines bacterial neutralization with controlled mucosal effector activity. Defining correlates of protection, optimal antigen combinations, appropriate mucosal adjuvants, and the durability of protective immunity remains a major research priority.

## 8 Knowledge gaps and future directions

Despite significant advancements in the understanding of the immune response to *H. pylori*, a number of obstacles hinder the conversion of the current data into a completely validated immunokinetic model. First, a large portion of the human literature is cross-sectional, which makes it challenging to ascertain whether identified immunological differences are temporary reactions to bacterial burden, consequences of established pathology, or causes of disease progression. To determine the temporal sequence of immunological events, longitudinal cohorts that start before or close to acquisition and continue via persistent infection, eradication, and post-eradication follow-up are therefore required.

Second, studies differ significantly in terms of patient age, sex, ethnicity, disease stage, bacterial genotype, anatomical sample site, specimen type, and analytical technique. Serum, plasma, gastric biopsy homogenates, tissue gene expression, immunohistochemistry, and ex vivo stimulation experiments are sometimes presented as similar biological measurements in cytokine investigations, which is especially troublesome. Before quantitative cytokine signatures may be assessed as repeatable biomarkers, standardized sampling, processing, normalization, assay platforms, and reporting systems are necessary.

Third, significant cellular and geographic variability within the affected gastric mucosa may be hidden by traditional bulk-tissue methods. The interaction of epithelial cells, neutrophils, macrophages, DCs, Th1/Th17 cells, Tregs, and B-cell populations inside particular gastric niches may be determined by single-cell RNA sequencing, spatial transcriptomics, multiplex tissue imaging, and integrated immune-receptor profiling. By combining these methods with bacterial genomics, it may be possible to learn more about how strain-specific traits like *cagA*, *vacA*, and adhesin profiles alter local immune architecture.

Fourth, further integration across biological scales is needed in this sector. Multi-omic techniques that integrate host transcriptomics, proteomics, metabolomics, epigenomics, microbiome profiling, and bacterial genomics may be able to differentiate immunological mechanisms that dictate persistence or disease progression from those that only correlate with inflammation. Clinically significant trajectories, such as asymptomatic persistence, ulcer illness, atrophy, intestinal metaplasia, and gastric cancer, should be the focus of such investigations.

Furthermore, there is still a lack of sufficient validation for predictive biomarkers. Future research should assess composite and longitudinal signatures that include cytokine patterns, immune-cell states, bacterial virulence traits, host genetics, histopathology, and microbiome characteristics rather than depending just on individual cytokine concentrations. Before these models can assist individualized risk assessment or therapeutic decision-making, they will need to be prospectively validated across spatial heterogeneity and ethnically varied populations.

When taken as a whole these gaps indicate that the immunokinetic framework’s central hypothesis that the timing, duration, anatomical location, and balance of inflammatory, regulatory, and bacterial adaptive processes may determine clinical outcome rather than the absolute magnitude of any one immune response remains to be tested prospectively. Longitudinal, systematic, multidimensional studies that can track host-pathogen interactions across time will be necessary to test this idea.

## 9 Conclusion

*H. pylori* persistence reflects dynamic interplay between tissue remodeling, immunological control, bacterial adaptability, and inflammatory activation. Available evidence supports indicates that innate, adaptive, regulatory, and humoral immunity exhibit recurrent but varying patterns; nevertheless, the extent, timing, anatomical location, and clinical implications of these patterns vary between persons and studies. Instead of creating a prospectively verified illness model, the suggested conceptual immunokinetic framework arranges these observations into a synthesis that generates hypotheses. To ascertain if the suggested temporal and spatial connections are repeatable and clinically significant, longitudinal investigations incorporating cellular, molecular, microbiological, and clinical data are necessary.
